# Surgical safety checklists for dental implant surgeries—a scoping review

**DOI:** 10.1007/s00784-022-04698-1

**Published:** 2022-08-27

**Authors:** Johannes Raphael Kupka, Keyvan Sagheb, Bilal Al-Nawas, Eik Schiegnitz

**Affiliations:** Department of Oral and Maxillofacial Surgery, Plastic Surgery, University Medical Centre of the Johannes Gutenberg-University, Augustusplatz 2, 55131 Mainz, Germany

**Keywords:** Implantology, Checklists, Dental surgery, Human factors

## Abstract

**Objectives:**

In both elective surgeries and aviation, a reduction of complications can be expected by paying attention to the so-called human factors. Checklists are a well-known way to overcome some of these problems. We aimed to evaluate the current evidence regarding the use of checklists in implant dentistry.

**Methods:**

An electronic literature search was conducted in the following databases: CINHAL, Medline, Web of Science, and Cochrane Library until March 2022. Based on the results and additional literature, a preliminary checklist for surgical implant therapy was designed.

**Results:**

Three publications dealing with dental implants and checklists were identified. One dealt with the use of a checklist in implant dentistry and was described as a quality assessment study. The remaining two studies offered suggestions for checklists based on literature research and expert opinion.

**Conclusions:**

Based on our results, the evidence for the use of checklists in dental implantology is extremely low. Considering the great potential, it can be stated that there is a need to catch up. While creating a new implant checklist, we took care of meeting the criteria for high-quality checklists. Future controlled studies will help to place it on a broad foundation.

**Clinical relevance:**

Checklists are a well-known way to prevent complications. They are especially established in aviation, but many surgical specialties and anesthesia adopt this successful concept. As implantology has become one of the fastest-growing areas of dentistry, it is imperative that checklists become an integral part of it.

## Introduction 

Treating a patient can be like a journey, the goal of which should be successful therapy, but unexpected twists and turns can occur along the way [1, 2]. Therefore, it is even more important to keep the patient’s safety in mind at all times and, if possible, to guarantee it. This analogy can be continued and specified: Many methods of aviation can be transferred to procedures in the operating room and can make an important contribution to quality management in the dental practice [3]. These include, for example, improved communication, recognition of fatigue, briefings, a blame-free culture, application of the sterile cockpit concept, or defined standards [4]. Thanks to strict adherence to these factors, air travel is considered the safest mode of transport [5]. In times before the structured organization of aviation, up to 70% of air accidents could be attributed to a lack of communication [6].

Treatment with dental implants in any form has been a growing branch of dentistry for years. In 2015/2016, the prevalence of dental implants in the USA was 5.7%, up from 0.7% in 1999. Projections expect it to rise as high as 23% in 2026 [7]. Therefore, it is a highly relevant issue not only for prosthodontists but also for oral- and maxillofacial surgeons. As an elective procedure, which allows for extensive planning, it is imperative that complications are prevented as far as possible.

Current research in implant dentistry is largely focused on technical and biological aspects of this field [3, 8, 9]. This includes, for example, developments regarding bone substitute materials [10–12], modifications of the implant surface [13–15], or platform switching [16–18]. The number of studies in this field is huge and systematic reviews and meta-analyses already exist on many of these issues [19–22].

Focusing exclusively on these factors could be a serious mistake, as studies show that only 4 out of 100 of the complications and errors are due to a lack of expertise [23]. Identifying typical sources of errors and systematically preventing them is therefore an indispensable part of modern medicine in both clinics and practices [24].

Checklists are a suitable instrument to increase the communication of the surgical team, consistently integrate defined standards into the daily routine, and establish reflective breaks [4, 25]. Other surgical specialties and anesthesia are already much more advanced in this area. Here, safety checklists are known to be an effective tool and are a widespread standard of quality assurance [26–29]. A systematic review published in the British journal of anaesthesiology by Abbott et al. included 11 studies with 453.292 surgical patients and investigated postoperative complications and mortality under the exposure of a surgical safety checklist. The results were a reduction in the complication rate (odds ratio: 0.73) and mortality (odds ratio: 0.75) [26]. Panesar et al. were able to demonstrate that even simple but severe complications like wrong site surgery can be reduced by over 20% [30].

Helmiö et al. conducted a study in the field of head and neck surgery. In that publication, the use of the checklist not only improved the communication of the surgical team significantly but also the awareness of the patient’s medical history and identity [31].

To our knowledge, there have been no reviews dealing with the topic of checklists in dental implantology. The aim was to clarify whether there are already drafts for adapted checklists and how they affect the therapy outcome, but also the compliance of the practitioners represents a relevant question.

## Methods

This review was designed and structured following the PRISMA checklist and statement.

### Focused questions

The following questions were formulated for the research. The aim was to clarify:

- whether checklists for implant therapy already exist in the literature,
- how high the acceptance and compliance of surgeons and practitioners are, and
- whether the use of checklists has a relevant influence on the treatment success.

Also, the following PICO-criteria were defined:

Population: Patients undergoing dental implant surgery.

Intervention: Surgical safety checklist.

Comparison: No checklist used.

Outcomes:Patient-related outcomes, complications.

Personnel-related outcomes, communication, compliance

### Search strategy

For this scoping review, an electronic literature search was conducted in the following databases: CINHAL, Medline (PubMed), Web of Science, and Cochrane Library (Cochrane Reviews). In addition, the reference list of included studies and related reviews was examined for relevant literature and studies. The last search was performed on March 15, 2022.The citation software Endnote 20 was used for collection and sorting (exclusion of duplicates and triplicates). The documentation of the searches was carried out with a commercially available software program (Microsoft Excel).

The author J. K. first searched the literature independently, which was followed by a review of the results by E. S. After removing duplicates, abstracts and titles were first checked for their relevance to the topic. In case of disagreement between the authors, the decision to include or exclude was made in a joint discussion. The used search terms are visualized in Table 1.

**Table 1:** Search terms

Dental implantology		Checklists
maxillofacial surger* OR oral surger* OR dental surger* OR oral implant* OR dental implant* OR operative dentist*	AND	checklist* OR standard operating procedure* OR sop* OR docket* OR worksheet* OR safe* checklist* OR mnemonic*

### Inclusion/exclusion criteria

We selected a wide range of studies for our scoping review to obtain as complete an overview as possible of the use of checklists in implant dentistry. Since only a small number of results was expected, a restriction was placed neither on the type of publications nor on the publication period. Consequently, both quantitative and qualitative papers were included. However, only English and German language literature was taken into account. Furthermore, all subheadings and MESH terms, as well as title and abstract, were searched.

Studies that dealt with oral surgery or maxillofacial surgery in general were excluded, although some of these are also mentioned in the discussion for example the study from Schmitt et al. [32]. Only studies that relate to dental implantology and in which a checklist was used or designed in any form to improve the outcome or for quality assurance were included.

## Results

The initial search yielded 1083 results, of which 269 could be excluded because they were duplicates or triplicates. After checking the title and abstract, a further 796 could be sorted out. Eighteen full articles were thus checked for compatibility with the inclusion criteria.

**Fig. 1:**
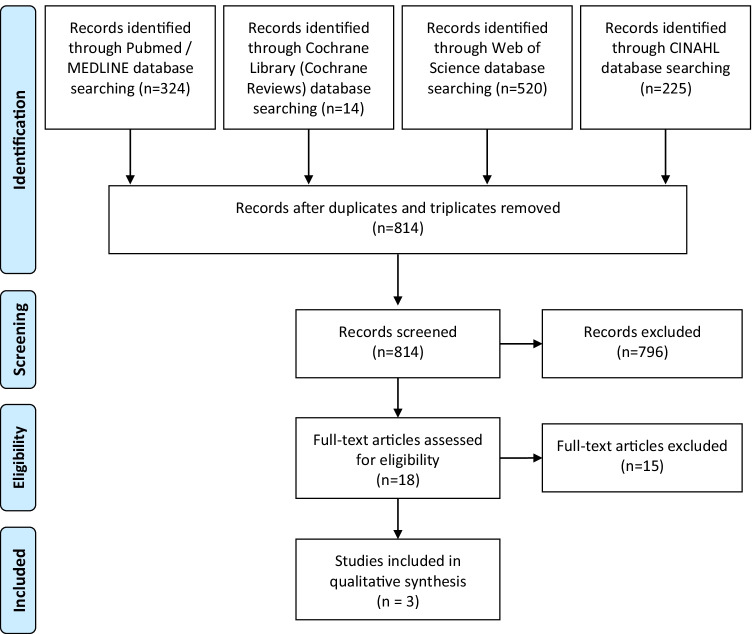
PRISMA flow diagram

Of these, only three met the above search criteria [33–35]. The others were not included for the following reasons: they were narrative reviews and no specific results could be presented regarding checklists in dental implantology (*n* = 5) [4, 36–39], the studies dealt with checklists in maxillofacial and/or oral surgery in general (*n* = 10) [32, 40–48]. Some of these studies fitted into both categories but are listed here only once. The PRISMA flow diagram in Fig. 1 illustrates the selection process.

One of the three publications dealt with the use of a checklist in implant dentistry and was described as a quality assessment study. The remaining two only offered suggestions for checklists based on a Delphi study or literature research and expert opinion. They were published between 2014 and 2021.

The quality assessment study examined the compliance of *n* = 8 prosthetic residents with the use of a surgical safety checklist with a total of 26 items (12 in the preoperative checklist and 14 in the postoperative checklist) in a single-center study. The checklist was used for 1 year at the University of Connecticut School of Dental Medicine. A total of 120 available surgeries were examined between 28.06.2017 and 28.06.2018 and the following items were recorded: number of implants inserted in one intervention, completed checklists, and items not completed on the checklists. There was no control group.

The checklist was used for all interventions, which corresponds to a compliance of 100%. Of all items that were requested (*n* = 120 × 26 = 3120), 2.4% were not answered. There was no correlation between the number of implants placed per surgery and the number of omitted items. The most frequent items not answered were preoperative photographs, postoperative analgesics, preoperative rinse with CHX, signed treatment plan, and the availability of radiographs [35].

### Suggestions for checklists

The proposed checklist by Bidra (also an author of the abovementioned quality assessment study) should be considered especially for the treatment of outpatients with varying degrees of difficulty and experience of the practitioners. It is not preceded by a systematic literature review and the points listed are, according to the author, partly evidence-based, but also based on experience from clinical practice. It is divided into three sections: preoperative, intraoperative, and immediate postoperative. Each section contains 12 to 14 items and two sections have sub-items. The order here is strongly oriented towards the clinical process [34].

In their publication, Christman et al. present a Delphi study to create a checklist for implant placement. A panel of 30 periodontists was formed who had at least 5 years of experience, were Diplomats of the American Board of Periodontology, and had already placed at least 1000 implants. In a total of three rounds, the most important points were first asked to the experts through an open question. In the second round, the points for which an agreement of at least 90% could be achieved were selected. Finally, a preliminary checklist was drawn up and re-evaluated by the participants. The result was a three-part checklist divided into a planning phase, an intraoperative phase, and a postoperative phase. Parts 1 and 3 each had 7 items, part 2 had 6. In the discussion of this publication, selected aspects are supported by a short literature review [33].

Comparing the checklists of the three studies, some similarities can be found. Since Bidra is the author of the quality assessment study as well as one of the mentioned checklist drafts, correspondence was to be expected. Remiszewski et al. used Bidra’s preoperative and postoperative checklist. The intraoperative one, on the other hand, was not taken into account.

But there are also similarities between Christman and Bidra. The following points were found in both publications:

- The assessment of the medical and dental history
- Reviewing the treatment plan as well as the patient’s signed consent
- Taking and reviewing preoperative radiographs
- Accurately assessing the position of the drill
- Prescribing antibiotics and antiseptic mouth rinses
- Making a follow-up appointment for the patient
- Giving postoperative instructions to the patient

Not only based on these two publications but also additional literature and the assessment of experienced practitioners from the University Hospital in Mainz, an own implant checklist is presented in the discussion section [8, 49–55].

## Discussion

### Overview

In general, the evidence regarding human errors in medicine is low [3]. It has now been shown that this trend continues in dental implantology (only *n* = 3 publications). However, this is in strong contradiction to the value attributed to checklists. In a systematic review that examined ways to improve patient safety in dentistry in general, checklists emerged as the only effective tool compared to reporting systems, trigger tools, and the use of electronic notes [48].

Schmitt et al. were already able to show the effectiveness of a checklist in oral surgery in a study of 80 surgeries (40 with and 40 without a checklist). More than four times as many complications occurred when no checklist was used. Similarly, there was a very high satisfaction rate among the participants, including dentists, surgeons, and nurses [32]. The cohort in this study was relatively small, but outside dentistry, the number of adverse events was reduced significantly even in larger collectives, as shown by Haynes et al. In this study, 7688 patients undergoing cardiac surgery were studied. After the implementation of a surgical safety checklist, the death rate reduced from 1.5 to 0.8% and the rate of complications from 11 to 7% [25]. Their value thus remains unquestionable.

Since the WHO has been providing a basis for more than 10 years with its surgical safety checklist, the lack of attention in implant dentistry remains particularly incomprehensible [56]. While there are further attempts to adapt this to general dentistry, its impact on implantology is still little researched [47], although even practitioners seem to be ready to use them. Helmiö et al. asked in their study whether surgeons would like to use a checklist, and 93% agreed [57].

However, a deficient understanding of the epidemiology of patient safety and quality assurance tools in dentistry might also be a reason for this. The patient’s perspective has also received little attention. Studies such as those by Wright et al. are pioneering [47]. With an error rate of about 2 per day, every practitioner should realize that human factors play a fundamental role in dentistry [58].

Nevertheless, checklists are not a panacea. The thoroughness of the users is of central importance. Routine is an enemy of the checklist [32]. Therefore, duplication of existing safety precautions must be avoided and medical staff has to be adequately trained in their use [59].

The time aspect also plays a role, especially considering the high pressure on costs in dental practices. For a single section, the practitioner should not need more than 1 min [47]. Possibly, such concerns can be dispelled after the implementation of the checklist. In a study by Kearns et al., 53% of respondents were concerned that the use of a checklist was inappropriate for emergency situations. After three months of use, this rate dropped to 30% [60].

### Critical examination of the included studies

The first publication presented here, by Bidra et al., is comparatively short at just over 600 words. It is a draft for an implant checklist that neither contains clear literature evidence nor defines the experts who contributed to its creation. Bidra himself is a prosthodontist [34]. With 12 or more items, the individual checklists tend to be too long [3]. Despite the weaknesses, we were able to take aspects of the work into account when creating our own checklist.

The second study dealt with the creation of a checklist using the Delphi method. The participating experts are interviewed separately on the topic of interest to prevent collusion and thus distortions [33, 61]. The size of the panel is based on similar studies in the field of dentistry and was relatively large, with thirty participating practitioners [62, 63]. This procedure reduces the effect of misjudgement by individuals and a broad spectrum of opinions is included. However, one disadvantage could be that the selection of experts has a considerable influence. In the present case, only periodontists were involved in the study. Thus, a bias in the setting of the focus can be expected, and it might be possible that influencing factors from other fields are not considered. This manifests itself, for example, in the fact that all members of the panel voted in favor of the periodontal status survey. In contrast, only 50% felt that the use of a surgical guide was important, and only 80% voted in favor of observing the correct drilling speed [33]. However, both points are found in Bidra’s draft [34, 35].

The retrospective single center study by Remiszewski et al. investigated how good compliance was with the use of a checklist in implantology. The framework conditions were clearly defined, but only 8 prosthetic residents were involved and only one pre- and one post-operative checklist was used. It would have been desirable to include a larger number of practitioners. In a multicenter study by Yu et al., 30,654 operations were examined in four hospitals. It turned out that compliance was not 100% as in the case of Remiszewski. Instead, the checklist was applied in the sign-in stage with an acceptable rate of 80.4–100%. In the time-out stage, it lowered to 40–88.8% and got even worse in the sign-out stage with only 10.2–59.5% acceptance [64]. It can therefore be assumed that a certain bias exists due to the small number of participants and the university environment in which the study of Remiszewski was conducted [35]. Since Bidra (also one author of the study) had already presented a three-part checklist in the aforementioned paper, it remains unclear why the intraoperative part was not evaluated and used [34].

There is no data on whether the checklist led to a reduction in complications. There is no before and after comparison, nor was a control group used without a checklist.

The fact that only few experienced practitioners were included also leaves out a crucial perspective. In the study by Krombach et al., it was shown that both particularly inexperienced and particularly experienced practitioners regarded checklists as useful. It can therefore be assumed that experience does not make checklists unnecessary but, on the contrary, the potential is appreciated even more [27]. In contrast, Visvanath et al. concluded by investigating oral and maxillofacial surgeons through questionnaires that particularly less experienced practitioners are willing to use a checklist and do so. However, as many as 42% of the respondents also stated that they do not use a checklist. This value is to be estimated all the higher since only 12% of the contacted practitioners participated in the survey and among them, the number of those interested in checklists could be disproportionately high [42].

As mentioned before, it would also be interesting to compare different implanting specialities (prosthodontists, periodontists, oral surgeons, maxillofacial surgeons, dentists).

Remiszewski et al. also take the temporal aspect to a certain extent into account, as a comparison of the operations is considered based on the number of implants inserted. Contrary to expectations, omissions were found more frequently in the groups with one or two implants. This is probably due to the different group sizes (1- or 2-implant surgeries *n* = 93 out of 120 total included surgeries) and should be re-evaluated in further studies [35]. A defined framework for the additional time required would be interesting, as in the study by Taylor et al. Here the checklist only took about 2 min [65]. Especially in dental practice, the question could arise whether this additional effort is tolerable. These concerns are shared by practitioners in many settings [59].

However, despite the weaknesses, Remiszewski provides the first study of checklists in dental implantology and thus makes a valuable contribution to this issue.

### A new surgical safety checklist for implant surgery

**Fig. 2:**
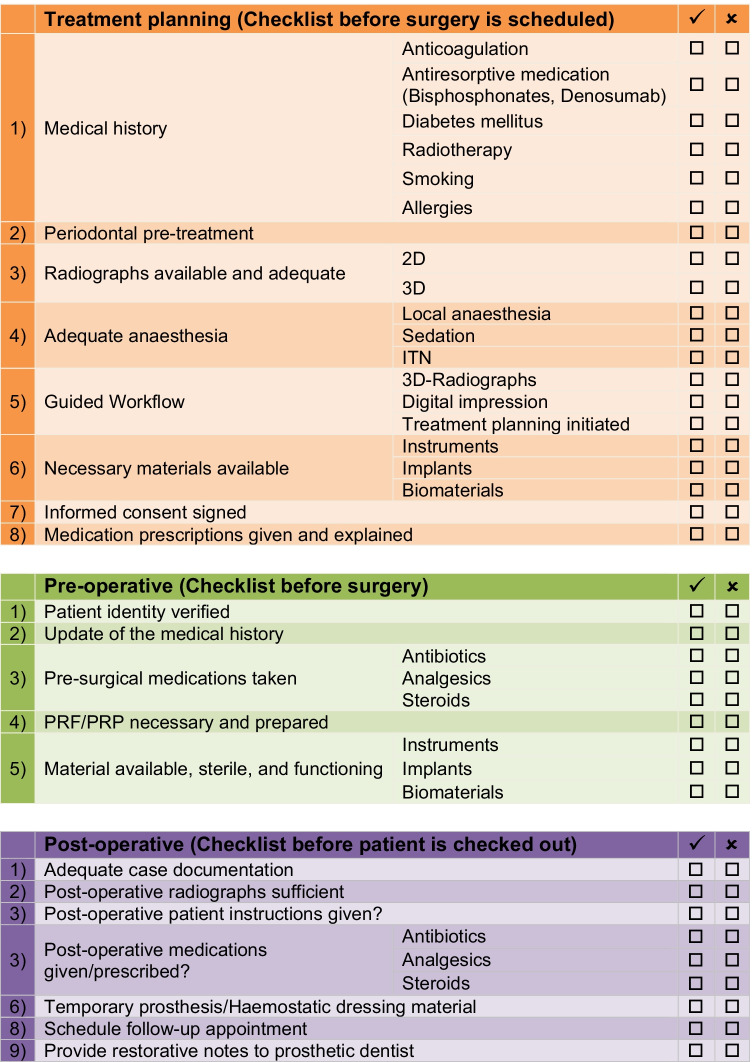
A new surgical safety checklist for implant surgery

In Fig. 2, the authors have designed their own checklist. The literature research of this review but also the experience and knowledge of maxillofacial surgeons and dentists of the Department of Maxillofacial Surgery of the University of Mainz were incorporated.

The individual points will not be explained in detail. A comprehensive description of the individual topics would go beyond the scope of this publication and, for this reason, cannot be considered useful. However, some characteristics of the formal design, which distinguish a high-quality checklist, should be mentioned:

First of all, it has been divided into three parts based on the typical treatment procedure of implantation: planning and information, preoperative, and postoperative [54, 55]. This promises complete, comprehensible quality management from the initial presentation of the patient to the completed implantation.

A significant amount of patient safety incidents in dentistry are avoidable pre- or postoperatively (62%). This is a result of the analysis of the National Patient Safety Agency database in the UK [66]. Therefore, we did not focus on the intraoperative period. Also, there might be huge differences between individual patients so the design of a universal checklist in this area seems not appropriate.

According to Renouard et al. and Gawande, the following factors are also important [3, 67].

We always chose fewer than 10 items. This keeps the checklists clear and they can be used very efficiently by the practitioner. If they become too detailed, important safety factors could be skipped and are over gone unnoticed.

It is also important that each item can be given a “no” rating. This might be the case, for example, because it does not apply to the patient in specific situations. This avoids items not being filled in and is especially useful when following up on an error. Otherwise, it remains unclear whether the item was forgotten in its entirety or simply not checked off: each of them requires an answer.

The checklist can be integrated into a digital patient file and serves to document the course of treatment. Nevertheless, the format also allows it to be used in paper form so that practices working with analog files are not excluded. The widely used and data protection–compliant PDF format is also compatible with nearly all mobile devices. Among others, Kiefel et al. already confirmed the advantages of digital working [68].

Despite the advantages, it may make sense to modify the checklists according to the preferences and needs of the practitioners.

## Conclusion

Overall, the data on checklists in dental implantology is rather weak. For this reason, we present three implant checklists in this paper to guide practitioners safely through the treatment process. Future studies will show how well these checklists can reduce complications and increase treatment success.

It will become one of the critical issues of dental implantology to make the advanced technical achievements even more successful by paying attention to the human factors. The use of checklists will be a central concept in preventing severe errors.

## Data Availability

The datasets used and/or analyzed during the current study are available from the corresponding author on reasonable request.
